# Ingestion of Insect Protein Isolate Enhances Blood Amino Acid Concentrations Similar to Soy Protein in A Human Trial

**DOI:** 10.3390/nu10101357

**Published:** 2018-09-22

**Authors:** Mathias T. Vangsoe, Rebekka Thogersen, Hanne C. Bertram, Lars-Henrik L. Heckmann, Mette Hansen

**Affiliations:** 1 Section for Sport Science, Department for Public Health, Aarhus University, Dalgas Avenue 4, 8000 Aarhus C, Denmark; mhan@ph.au.dk; 2 Department of Food Science, Aarhus University, Kirstinebjergvej 10, 5792 Aarslev, Denmark; rebekka.thoegersen@food.au.dk (R.T.); hannec.bertram@food.au.dk (H.C.B.); 3 Danish Technological Institute, Life Science Division, Kongsvang Allé 29, 8000 Aarhus C, Denmark; lhlh@teknologisk.dk

**Keywords:** alternative protein source, nutrition, NMR spectroscopy, metabolomics, lesser mealworm, postprandial absorption, amino acid availability

## Abstract

Background: Increased amino acid availability stimulates muscle protein synthesis (MPS), which is critical for maintaining or increasing muscle mass when combined with training. Previous research suggests that whey protein is superior to soy protein in regard to stimulating MPS and muscle mass. Nevertheless, with respect to a future lack of dietary protein and an increasing need for using eco-friendly protein sources it is of great interest to investigate the quality of alternative protein sources, like insect protein. Objective: Our aim was to compare the postprandial amino acid (AA) availability and AA profile in the blood after ingestion of protein isolate from the lesser mealworm, whey isolate, and soy isolate. Design: Six healthy young men participated in a randomized cross-over study and received three different protein supplementations (25 g of crude protein from whey, soy, insect or placebo (water)) on four separate days. Blood samples were collected at pre, 0 min, 20 min, 40 min, 60 min, 90 min, and 120 min. Physical activity and dietary intake were standardized before each trial, and participants were instructed to be fasting from the night before. AA concentrations in blood samples were determined using ^1^H NMR spectroscopy. Results: A significant rise in blood concentration of essential amino acids (EAA), branched-chain amino acids (BCAA) and leucine was detected over the 120 min period for all protein supplements. Nevertheless, the change in AA profile was significantly greater after ingestion of whey than soy and insect protein (*p* < 0.05). Area under the curve (AUC) analysis and AA profile revealed comparable AA concentrations for soy and insect protein, whereas whey promoted a ~97% and ~140% greater AUC value than soy and insect protein, respectively. A tendency towards higher AA concentrations beyond the 120 min period was observed for insect protein. Conclusion: We report that ingestion of whey, soy, and insect protein isolate increases blood concentrations of EAA, BCAA, and leucine over a 120 min period (whey > insect = soy). Insect protein induced blood AA concentrations similar to soy protein. However, a tendency towards higher blood AA concentrations at the end of the 120 min period post ingestion was observed for insect protein, which indicates that it can be considered a “slow” digestible protein source.

## 1. Introduction 

Protein is an essential macronutrient and is highly important for building skeletal muscle structure by providing nitrogen and amino acids [1]. Furthermore, amino acid availability is known to stimulate muscle protein synthesis (MPS) which is necessary for the accretion of skeletal muscle mass [2]. Research indicates that a daily habitual protein intake of 0.8–1.6 g/kg/d [1,3] is sufficient to maintain a positive net protein balance and avoid loss of muscle mass, which is of great relevance for the majority of the population, but especially the elderly with low daily energy intake. 

The world population is constantly growing and is projected to reach 9.8 billion people by 2050 [4]. Therefore, the demand for sustainable and nutrient-rich food seems of vital importance. The United Nations Food and Agriculture Organization (FAO) estimates that the global food production must increase >70% by 2050 to feed the growing world population [5]. This fact highlights the importance of identifying new and sustainable animal-based foods that provide a rich source of protein for human nutrition. Animal-based foods with a high protein content such as meat, eggs, and milk are all considered as high-quality protein sources because of their attractive amino acid composition and high digestibility [6]. Protein from insects has the potential of being an eco-friendly, high-quality solution to meet future protein demands, and literature suggests that some insect proteins are equivalent or superior to soy protein in terms of nutritional value [7,8]. However, the nutritional value of this protein source is only very scarcely investigated and especially human studies are lacking [8,9]. 

Maintaining or increasing muscle mass is of great importance for both frail elderly and sports athletes. Protein is an essential macronutrient and is highly important for building skeletal muscle structure by providing nitrogen and amino acids [1]. Furthermore, a high amino acid availability has a stimulating influence on muscle protein synthesis (MPS) which is necessary for the accretion of skeletal muscle mass [2]. A meta-analysis by Cermak et al., 2012 [10] reports that protein supplementation increases muscle mass and strength during prolonged resistance-type exercise. However, in a recent study, we found that supplementation of insect protein isolate enhances muscle mass and strength gains in young men during progressive resistance training, but with no significant difference to an isocaloric carbohydrate supplement [11]. We anticipate that a sufficient habitual protein intake in both groups revokes the potential effect of protein supplementation during resistance training in accordance with recent research [10,12]. Alternatively, the explanation for a lack of effect of insect protein on gain in muscle mass during training could be explained by the quality of the insect protein regarding bioavailability, digestibility, and amino acid (AA) profile. Protein quality mostly refers to the concentration of the nine essential amino acids (EAAs) as EAA is found to be the key promotor for MPS. Some studies have indicated that ingestion of EAAs alone stimulate MPS to the same extend as a whole protein with the same EAA content [13]. Furthermore, branched-chained amino acids (BCAAs) leucine, isoleucine, and valine have been found to be crucial for protein metabolism, blood glucose, and insulin regulation [14]. In particular, leucine alone has been suggested to further increase the peak MPS [15], however, a high total dose of EAAs still seems most suited for a sustained increase of MPS [16]. While several studies have been conducted on protein quality of whey, soy, and casein [2,17,18,19], an evaluation and comparison in concentration of EAAs and BCAAs in insect protein would be of great interest. Furthermore, previous research has found that products containing animal-based proteins contains the highest amount of EAAs compared to vegetable-based proteins [2,12], which give rise to a comparison of insect, whey, and soy protein, while these represent both animal- and vegetable-base proteins.

As no studies have identified AA bioavailability on pure insect protein isolate, the aim of the present study was to compare the AA profile and AA bioavailability of insect protein isolate, whey isolate and soy isolate. We hypothesized that insect protein isolate contains a higher amount of EAA, BCAA and has a higher bioavailability of AAs compared to soy protein isolate since it is an animal protein source. Furthermore, we expected that whey protein isolate would promote superior concentrations of EAA, BCAA, and leucine than soy and insect protein isolate, as supported by previous research [2,19,20,21]. 

## 2. Method 

### 2.1. Subjects

Six healthy young males, between 18 and 30 years of age, were recruited from the local Department for Public Health at Aarhus University, Section for Sport Science. All participants were screened against a standard criteria before inclusion, and group characteristics are shown in Table 1. Exclusion criteria were smoking, medication, or health issues conflicting with the experimental trial. The trial complied with the Declaration of Helsinki and was approved by The Central Denmark Region Committees on Health Research Ethics (Journal 60186, case number 1-10-72-320-16). All subjects gave their informed consent to participate before the experiment was carried out.

### 2.2. Experimental Protocol 

**Figure 1:**
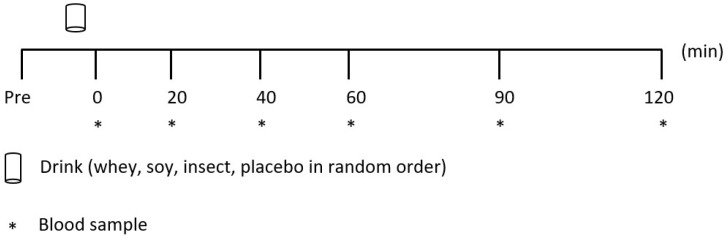
Experimental protocol.

The experimental trial consisted of four days with a protein supplementation in random order (25 g of crude protein from either whey isolate, soy isolate, insect protein isolate, or placebo (water). Blood samples were obtained for two hours following ingestion (Figure 1) and analyzed by ^1^H NMR spectroscopy to determine the changes in blood concentration of AA. 

Subjects arrived at the laboratory on the morning of each experimental trial after an overnight fast. To ensure dietary and physical activity standardization before each trial, subjects recorded dietary intake and physical activity level questionnaire the day before the first trail, which served as a standard plan before the following three experimental days. After consuming a drink containing 25 g of crude protein (~100 kcal) either as whey isolate (28.7 g powder), soy isolate (27.8 g powder), insect isolate (30.5 g powder), or placebo (0 kcal) dissolved in 400 mL water, blood samples were drawn at fixed times points the following two hours. The subject remained calm in a resting position during the two hours trial. All experimental trials were performed at the Section for Sport Science, Department for Public Health at Aarhus University.

### 2.3. Protein Supplement

Three type of protein isolate powder were tested (whey, soy, and insect protein isolate). Whey protein isolate (Lacprodan^®^ SP-9225 Instant, Arla Foods Ingredients, Viby, Denmark) (87% protein, 357 kcal/100 g, 22.7 g BCAA, 55.3 g EAA) was provided by Arla Foods Ingredients P/S (Viby, Denmark) Soy isolate (90% protein, 400 kcal/100 g) was provided by LinusPro Aps (Aarhus, Denmark). Insect protein isolate (Experimental Protein Isolate, ~82% protein) from the lesser mealworm (*Alphitobius diaperinus*) was provided by Proti-Farm R&D BV (Ermelo, Netherlands). Protein powder corresponding to 25 g crude protein (28.7 g of whey, 27.8 g of soy or 30.5 g of insect protein) was dissolved in 400 mL water. No taste or sweetening was added and none of the products contained any flavoring. Subjects were given a nose clip, to blind for the taste of the beverage. 

### 2.4. Blood Samples 

On each experimental trial, seven blood samples of ~8 mL were collected into evacuated containers containing lithium heparin. Before the subjects consumed the protein beverage, a baseline sample was taken (Pre), after which they consumed the intervention beverage within < 1 min. Blood samples were collected at 0 min, 20 min, 40 min, 60 min, 90 min, and 120 min after ingestion of the intervention beverage [12]. We would expect blood AA concentrations to have reached peak and decrease within this 120 min period as detected in previous studies [2,22]. The blood samples were centrifuged at 1200× *g* for 10 min at 4 °C to separate the plasma and stored at −80 °C until further analysis. Serum insulin concentrations were analyzed using a Roche Cobas e601 (Roche, Mannheim, Germany) at Aarhus University Hospital, Denmark. 

### 2.5. Sample Analysis Preparation

Plasma samples were thawed and filtered using 0.5 mL 3K Amicon Ultra centrifugal filter units (Merck Millipore Ltd., Cork, Ireland). Prior to use, the filter units were subjected to washing three times with MilliQ water (Merck KGaA, Darmstadt, Germany). For filtering, 500 µL plasma was transferred to the filter units and centrifuged at 4 °C, 14,000× *g* for 1 h. Another 200 µL plasma was added to the filters followed by centrifugation at 4 °C, 14,000× *g* for 30 min. A volume of 480 µL plasma filtrate was transferred to a 5 mm NMR tube containing 60 µL phosphate buffer (50 mM phosphate buffer with 1 mM 3-(Trimethylsilyl)-1-propanesulfonic acid-d6 sodium salt (DSS; Sigma-Aldrich, St. Louis, MO, USA) in final volume) and 60 µL deuterium oxide (D_2_O). All samples were analyzed in replicates with a total of 336 samples. 

For analysis of protein samples 100 mg whey, soy or insect protein was dissolved in 1.5 µL MilliQ water and centrifuged at 4 °C, 14,000× *g* for 5 min. The supernatant was filtered and analyzed by ^1^H nuclear magnetic resonance spectroscopy (^1^H NMR) using the same procedure and parameters as described for the plasma samples. 

### 2.6. ^1^H NMR Spectroscopy

^1^H NMR spectroscopy was conducted using a Bruker Avance III 600 MHz spectrometer operating at a ^1^H frequency of 600.13 MHz with a 5 mm ^1^H TXI probe (Bruker BioSpin, Rheinstetten, Germany). Spectra were obtained at a temperature of 298 K using a ^1^D nuclear overhauser enhancement spectroscopy (NOESY)-presat pulse sequence (noesypr1d) to suppress the water signal. The acquisition parameters used were: 128 scans (NS), spectral width (SW) = 7211.54 Hz (12.02 ppm), acquisition time (AQ) = 2.27 s, 32768 data points (TD), relaxation delay (D1) = 5 s. The free induction decays (FIDs) were multiplied by a line-broadening function of 0.3 Hz prior to Fourier transformation. The spectra obtained were baseline and phase corrected using TopSpin 3.0 (Bruker BioSpin, Rheinstetten, Germany). Metabolite assignment and quantification was conducted using Chenomx NMR Suite 8.13 (Chenomx Inc., Edmonton, Alberta, Canada).

### 2.7. Amino Acid (AA) Profile and Analysis 

All AA analyses of the protein supplements were performed by Eurofins Steins Laboratory A/S (Vejen, Denmark). Samples were hydrolyzed in aqueous hydrochloric acid. After hydrolysis, the samples were pH-adjusted (pH 1.0–2.5), brought to volume with loading buffer (pH 2.2) and filtered. Amino acids were separated in an amino acid analyzer and the detection was carried out using post column derivatisation with ninhydrin reagent and 440 and 570 nm. For quantification, a one-point calibration was used, with a 0.0 comparison range to a known reference concentration. For quality assurance, an in-house standard, pet food, was analyzed in every run. All analyses met the criteria of EURL 152/2009 and ISO 13903:2005. 

### 2.8. Statistics 

Statistical analyses were performed using the EpiBasic 3.0 (version 3.0, 2013. Svend Juul and Morten Frydenberg, Aarhus, Denmark). All blood sample concentrations were analyzed using a two-factor repeated-measures ANOVA. All data were tested for normality of distribution, and for data not meeting this, Gubbs’ test was conducted to detect outliers in the data which were then excluded from the analysis. Area under the curve (AUC) data were analyzed using a two-factor repeated-measures ANOVA. Statistical significance was defined as *p* < 0.05. All data are presented as means ± SD. 

## 3. Results

Blood AA concentrations were analyzed of which EAA, BCAA, leucine, and area under the curve (AUC), were evaluated. The analyses evaluated blood concentrations of histidine, isoleucine, leucine, lysine, methionine, phenylalanine, threonine, and valine, with no detection of tryptophan. All samples were replicated, and with a total of 336 blood samples 11 samples were lost during the analysis, with six samples representing the placebo trail. 

**Figure 2:**
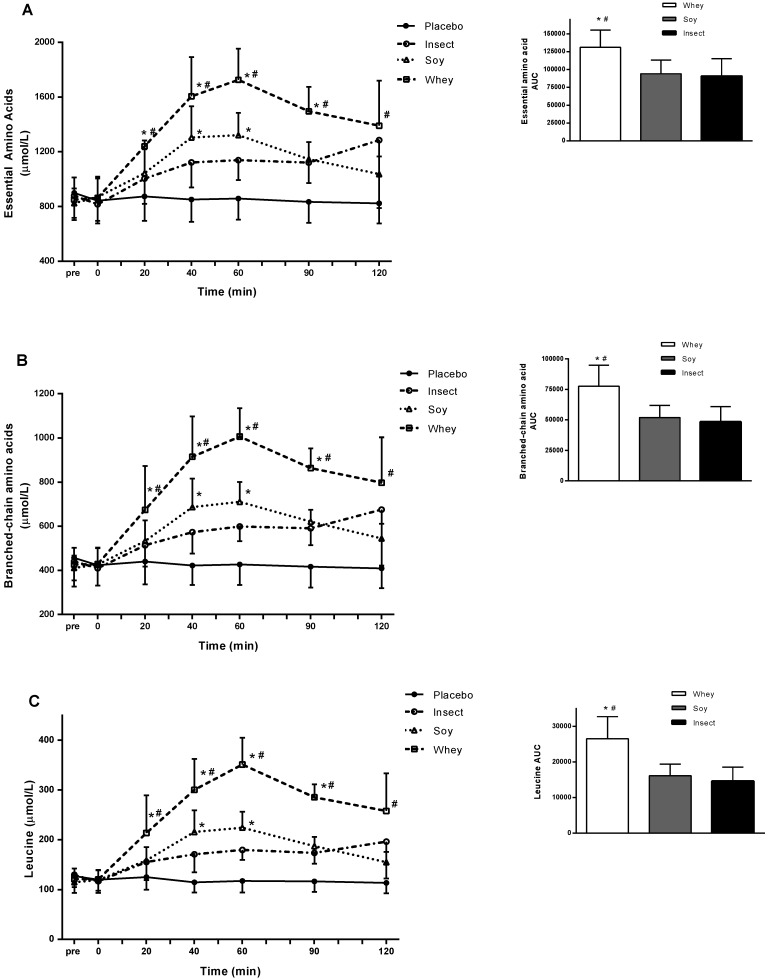
Blood concentrations of essential amino acids (**A**), branched-chain amino acids (**B**), and leucine (**C**) after ingestion of whey, soy, or insect protein isolate. Insert: area under the curve (AUC) analysis for each concentration evaluated as incremental AUC from time = 0 min. * Significant different from insect protein (*p* < 0.05). # Significant different from soy protein (*p* < 0.05). All data was significant different from placebo at all time-point after 0-min. All values are mean ± SD; *n* = 6 per group.

**Figure 3:**
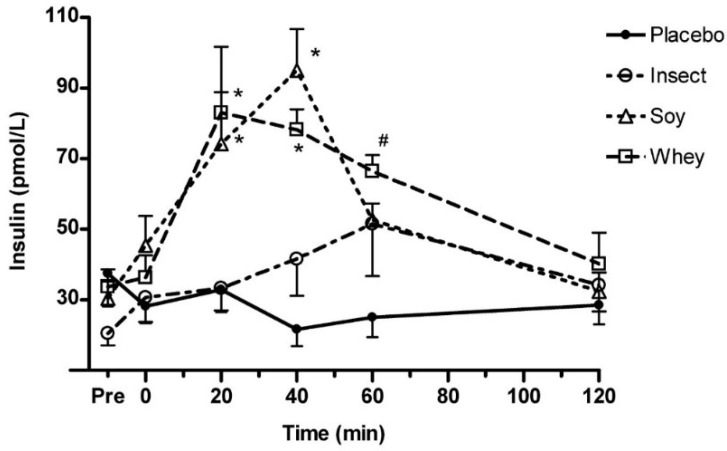
Serum insulin concentration after ingestion of whey, soy, insect, or placebo. Blood samples were obtained pre and 0, 20, 40, 60, and 120 min post-prandial. * Significant different from insect isolate (*p* < 0.05), # significant different from soy isolate for same condition (*p* < 0.05). All values are mean ± SD; *n* = 6 per group.

Data showed a significant higher AA concentration in all trails with protein ingestion compared with placebo (*p* < 0.05), and all measures followed the same general pattern. Whey protein stimulated a significantly greater concentration of EAA (Figure 2A), BCAA (Figure 2B), and leucine (Figure 2C) compared with soy and insect protein ingestion (*p* < 0.05). At 60 min post consumption, the blood concentrations of EAA, BCAA, and leucine after both whey and soy protein ingestion peaked. In contrast, the highest amino acid blood concentrations after insect protein ingestion were detected at 120 min post consumption with a tendency towards greater leucine concentration compared with soy at the time-point 120 min (*p* = 0.073) (Figure 2C). AUC analysis revealed EAA to be ~97% greater after whey protein consumption compared with soy protein (*p* < 0.01 whey vs. soy), and ~140% greater than insect protein (*p* < 0.01 whey vs. insect). Blood levels (AUC) of EAA, BCAA, and leucine did not differ significantly between soy and insect protein (Figure 2A–C). Serum insulin concentrations increased significantly 20 and 40 min postprandial in the whey and soy protein trial compared to insect protein (*p* < 0.05) (Figure 3). At 60 min the insulin concentration remained high in the whey protein trial compared with soy isolate (*p* < 0.05). Insulin concentration was significantly higher after ingestion of either whey or soy protein compared to placebo at 20–60 min (not marked on figure). Insect protein did not differ from placebo, however, a tendency towards higher insulin at 40–60 min were observed (*p* = 0.08; *p* = 0.09, respectively). During the analysis 11 of 94 data points were excluded after conducting Gubbs’ test for outliers. 

**Figure 4:**
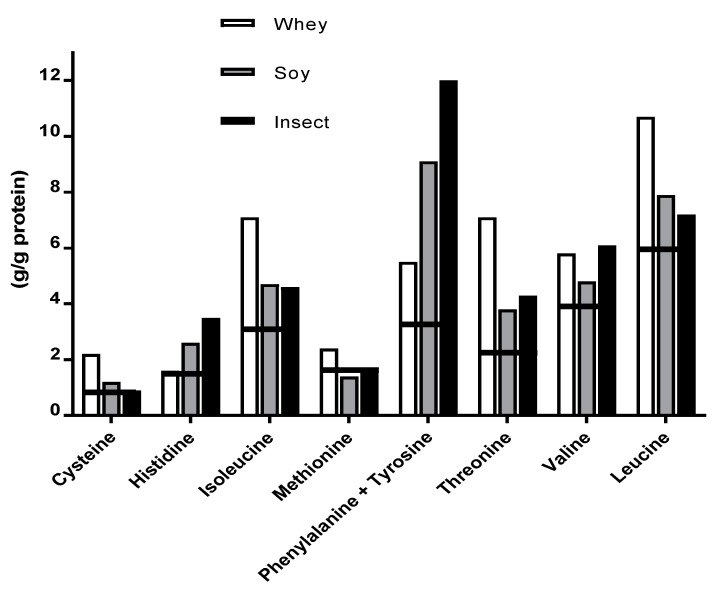
Amino acid content of pure protein shown in comparison to the FAO’s current adult requirement (horizontal lines). All values are presented as means, see Table 2.

Evaluation of AA profiles in the different types of protein supplements revealed total amino acid (TAA) content of 96.2%, 83.2% and 69.1% for whey, soy and insect respectively (without tryptophan) (Table 2). EAA content for all three protein sources met the FAO/WHO/UNU [23] requirements (27.7 g/g protein) for adults. In addition, whey and insect protein met all requirements for each EAA, while soy protein did not meet the requirements of methionine (1.6 g/g protein), (Figure 4).

## 4. Discussion 

This is the first study to investigate the postprandial response after ingestion of insect protein isolate on AA blood concentrations in vivo in humans. Furthermore, it is the first time a direct comparison between whey, soy, and insect protein isolate has been performed to evaluate and compare the AA profile and AA availability after ingestion in a human experiment.

The purpose of this study was to examine the bioavailability of AAs over 120 min post protein ingestion, identify and compare the AA profile of whey, soy, and insect protein, and thereby clarify the potential of insect protein as an alternative dietary protein to meet the future rise in food demands.

We found that ingestion of either whey, soy or insect protein isolate all resulted in a significant increase of EAA, BCAA and leucine compared to placebo (water), however, ingestion of whey protein isolate led to significantly higher concentrations of AAs compared with soy and insect protein. Despite the fact that ingestion of soy protein increased blood EAA, BCAA, and leucine concentrations significantly at 40 and 60 min (Figure 2A–C) AUC analysis revealed no significant difference between soy or insect protein measured over the 120 min period (Figure 2). This finding is partly due to a slow release of AAs after ingestion of insect protein, which is mostly expressed by the end of the test period. Similarly, evaluation of the AA profile showed comparable EAA and BCAA content for soy and insect protein. This fact suggests that differences in blood AA concentrations between soy and insect protein is due to a slower digestion of the insect protein. Furthermore, a significant increase of serum insulin was detected after ingestion of whey and soy protein, while insect protein did not seem to affect serum insulin concentration to the same extent.

Our findings support previous evaluation of whey protein as a high-quality “fast digestible” protein source [2,19,21,24] compared with soy protein. Furthermore, the blood AA profile post whey ingestion is in agreement with previous studies [2,21]. Nevertheless, as this is the first study comparing ingestion of whey, soy or insect protein, a novel observation was that insect and soy protein promoted a comparable rise in blood AA concentrations measured over the first two hours postprandial. This is further in line with previous studies evaluating and comparing the nutritional values of insects [8,25], however our data do not support insect protein to be superior to soy as suggested by Finke et al., 1989 [7]. The incongruence between these findings and our hypothesis as insect protein being of higher quality than soy could be due to: 1) differences in analytical methodologies for quantification of plasma AAs; 2) high concentrations of insoluble fibers present in the insect protein supplement, and solubility of the insect protein; 3) deviations in determining the proteins’ AA profile; and 4) the period measuring blood concentration of AAs.

As this is the first study to investigate in vivo postprandial ingestion of insect protein, the literature on this topic is very limited. The majority of previous research is based on in vitro assessments of insect protein [8,25,26], which must be considered a substantial limitation when evaluating potential alternative food sources for human nutrition. Thus, most of the former studies recommend future approaches for protein quality to be evaluated in humans, as conducted in the present study.

A recent study by Churchward-Venne et al., 2017 [25], evaluated insect protein as a protein source for human consumption and found that the digestibility and AA profile of the protein could be a limiting factor when considering its nutritional value. Churchward-Venne et al., 2017 [18] argue that a high content of chitin, a nitrogen-containing polysaccharide mainly found in the insects’ exoskeleton, and insoluble fibers may affect the digestibility of the protein. Furthermore, earlier studies have reported termite protein to be less soluble than casein (51% vs. 84%, respectively) when assessed in vivo in rats [27]. Such a lower solubility could partly contribute to lower concentrations of AAs as detected in the present study, whereas we would expect animal-based protein sources to be more digestible than plant protein sources [6,21]. Therefore, to further contribute to our understanding of its digestibility, a future evaluation of fiber content in insect protein products and the digestion of these would be of great interest. This could beneficially be supported by a digestible indispensable amino acid score (DIAAS) for different insect protein sources [28].

Our secondary aim was to determine the AA profile of protein powder products and compare these with blood AA concentrations and findings in previous studies. The analysis detected higher TAA, EAA, and BCAA values for whey protein than soy and insect, however, no notable differences were detected between soy and insect protein powder. Existing literature reporting AA profiles and EAA content of whole insects and refined insect protein is somewhat equivocal. Studies have reported the sum of EAA in insects to be comparable to soy protein and superior to casein [7,8] and, furthermore, to meet adult requirements presented by the FAO [23]. Contrarily, more recent studies have found the EAA content of insects to be low and unable to meet the adult requirements by the FAO [25,27]. Variations may be explained by a lack of standardization of diet or feed, variation between insect species, and analytical methodologies [8]. Moreover, several studies have reported a potential risk of overestimating the true protein content due to a high nitrogen content in the insects’ exoskeleton [8,25]. In our study, we detected a discrepancy between presumed TAA content of soy (90%), insect (82%), and whey (87%) protein declared by the producer, and the TAA determined when analyzing the protein supplement (83.2%, 69.1%, and 96.2%), respectively. This divergence may have an impact on our evaluations and comparison of blood AA concentrations. Nevertheless, if adjusted for variation of dose, ingestion of EAA and BCAA would have been almost identical for soy and insect protein (9.2 g, 3.9 g vs. 9.5 g, 3.7 g). However, we acknowledge that differences in presumed TAA and the evaluated TAA has created a small difference between the intended dose of protein supplementation and the actual dose given. If we recalculate the amount of protein actually ingested by the participants, they consumed 27.6 g whey protein, 23.2 g soy protein, and 21.1 g insect protein, respectively. If the total blood concentration of EAA is adjusted and calculated as AA per gram crude protein, the data would be 4750 μM EAA/g whey protein, 4051 μM EAA/g soy protein and 4303 μM EAA/g insect protein. Similarly, the adjusted data for leucine would be 960 μM leucine/g whey protein, 694 μM leucine/g soy protein and 693 EAA μM/g insect protein. This would translate into a difference of only ~10% in EAA between whey and insect protein isolate, and a ~6% higher EAA concentration for insect compared to soy. The same pattern is evident in leucine concentration with a ~3% greater concentration for whey isolate, but no difference between soy and insect protein. This means that the difference between whey and insect protein becomes notably smaller, and that our original evaluation therefore may have underestimated the results as first noted. A figure of adjusted values is shown in supplementary material (Figure 5). 

A tendency towards higher AA plasma concentration for insect protein compared with soy were detected, as AA concentrations were steadily increasing until last time point at 120 min (leucine: *p* = 0.07; EAA: *p* = 0.11; BCAA: *p* = 0.10) insect vs. soy respectively. This finding may reflect that insect protein would induce even higher AUC values compared with soy, if measured over a longer period (> 120 min). A blood sampling period for 3–4 hours would have given a more fulfilled picture of the digestibility of the protein sources. However, it should be noted that the AA content of the soy and insect protein powder was comparable (Table 1). Therefore, our results suggest insect protein to be more slowly digested than soy. 

Another novel finding of this study would be the differences in insulin response after ingestion of the protein supplementations. Ingestion of whey or soy protein induced a significant increase in serum insulin concentration at 20–40 min post-ingestion compared to insect protein. This was somehow a surprising finding as we would have expected insect protein to stimulate a similar increase in serum insulin. However, the response seems somewhat similar to previous evaluations of insulin response after ingestion of casein which, furthermore, has a similar slower digestion as found for insect protein [2,29]. This indicates that while insect protein partly induces a similar increase in blood EAA concentration as soy protein, the digestion and insulin response is markedly slower and more moderate as the characteristics of casein. While some studies suggest the protein stimulated increase of insulin to further increase MPS, a study by Fujita et al., 2006 [30] evaluates that blood flow and AA availability is the most pronounced condition to stimulate MPS. However, it would be relevant for future research to investigate the response on MPS after ingestion of either whey, soy or insect protein.

The potential of insect protein as an exercise and sports nutrition supplement can be opposed, based on the findings in our recent study [11]. After exercise, a rapid increase in EAA, leucine, in particular, is important for supporting maximal rates of skeletal MPS [2,31,32,33]. The insect protein isolate extracted from lesser meal worms, which was tested in the present trial, may not meet this requirement. In contrast, it is well-documented that whey protein is a high-quality source and easily digested as documented in the present trial. In line with this, whey protein induces a significantly larger increase in muscle MPS than soy and casein [2]. Further, it has been suggested that “fast” proteins like whey stimulate a rapid rise in MPS, while “slow” proteins like casein, and probably also insect protein, primarily seems to inhibit muscle protein breakdown [29]. Ingestion of whey protein combined with resistance training, therefore, still appears to be the most optimal combination to support muscle growth and strength gains as desired for athletes.

Considering insect protein as a dietary protein supplement in elderly to prevent age-associated muscle loss could still be of relevance. However, specific studies on this group of subjects are still needed to evaluate the potential of the protein in the prevention of sarcopenia. Some previous studies have found that “fast” leucine-rich proteins have the greatest benefits in stimulating MPS in elderly both at rest and after resistance exercise [34,35], which may oppose the use of insect in this coherence. However, other works suggest that prevention of muscle loss in the elderly may also be influenced by other factors and that ingestion of a diet rich in the amino acid glycine can restore MPS during aging and immobilization [36]. Noticeably, the glycine content is higher in soy and insect protein than whey (Table 2). However, glycine content is yet substantially higher in collagen protein hydrolysates [37].

As the literature on the true protein content and sum of EAA varies, it remains uncertain whether insect protein could supersede soy protein as a high-quality eco-friendly protein for human ingestion. Nevertheless, based on our findings in the present study, insect protein could at least match the quality of soy protein, however, it is yet “slowly” digested. This would, therefore, suggest insect protein to be a potential nutritional compensation for meat, egg, and soy-based foods, which serve as protein rich food sources in common human diets.

In summary, we report that ingestion of whey, soy and insect protein isolate increases blood concentrations of EAA, BCAA and leucine over a 120 min period (whey > soy = insect). Insect protein induced blood AA concentrations similar to soy protein over a two-hour period post-prandial, but seems to be more slowly digested than both soy and whey. Still, the data suggests that protein extracted from insects may be a potential future alternative to soy protein in daily human nutrition.

## Figures and Tables

**Table 1:** Subject characteristics.

Subjects (*n* = 6)	
Age (y)	24 ± 1
Height (cm)	183 ± 6
Weight (kg)	81 ± 12
Activity level (h)	11 ± 4

y, years; h, hours of physical activity per week. All values are mean ± SD.

**Table 2:** Total amino acid content (TAA), branched-chain amino acid (BCAA), essential amino acid (EAA). Amino acid content evaluated as AA g/100 g and AA g/g protein. Tryptophane content was not measured.

Amino Acid	Whey g/100g	Protein g/g	Soy g/100g	Protein g/g	Insect g/100g	Protein g/g
Alanine, g	5.2	5.4	3.5	4.2	5.1	7.4
Arginine, g	2.0	2.1	6.2	7.5	4.0	5.8
Asparagine, g	10.0	10.4	10.0	12.0	6.4	9.3
Cycteine (Cystin), g	2.1 ^a^	2.2	1.0	1.2 ^a^	0.6	0.9 ^a^
Glutamine, g	17.3	18.0	16.2	19.5	10.4	15.1
Glycine, g	1.4	1.5	3.5	4.2	3.4	4.9
Histidine, g *	1.5	1.6 ^a^	2.2	2.6 ^a^	2.4	3.5 ^a^
Isoleucine, g *^,^ #	6.8	7.1 ^a^	3.9	4.7 ^a^	3.2	4.6 ^a^
Leucine, g *^,^ #	10.3	10.7 ^a^	6.6	7.9 ^a^	5.0	7.2 ^a^
Lysine, g *	9.1	9.5	5.3	6.4	4.6	6.7
Methionine, g *	2.3	2.4 ^a^	1.2	1.4	1.1	1.6 ^a^
Phenylalanine, g *	2.7	2.8 ^a^	4.4	5.3 ^a^	3.1	4.5 ^a^
Proline, g	6.0	6.2	4.4	5.3	4.2	6.1
Serine, g	4.5	4.7	4.4	5.3	3.1	4.5
Threonine, g *	6.8	7.1 ^a^	3.2	3.8 ^a^	3.0	4.3 ^a^
Tyrosine, g *	2.6	2.7 ^a^	3.2	3.8 ^a^	5.2	7.5 ^a^
Valine, g *^,^ #	5.6	5.8 ^a^	4.0	4.8 ^a^	4.2	6.1 ^a^
TAA, g	96.2	100.0	83.2	100.0	69.1	100.0
# BCAA, g	22.7	23.6	14.5	17.4	12.4	18.0
* EAA, g	47.2	49.1 ^a^	31.8	38.2 ^a^	27.3	39.4 ^a^

#, a part of total BCAA. *, a part of total EAA.a Meeting FAO/WHO/UNU [23] requirements for adults’ protein intake (g/g. protein) regarding (Histidine, Isoleucine, Leucine, Lysine, Methionine, Cysteine, Phenylalanine, Tyrosine, Threonine, Valine).

## Supplementary Materials

The following are available online at http://www.mdpi.com/2072-6643/10/10/1357/s1, Figure 5: Adjusted blood amino acid concentrations of essential amino acids (EAA), branched-chain amino acids (BCAA) and Leucine after ingestion of whey, soy or insect protein isolate.
