# Supplementary material for: Ingestion of Insect Protein Isolate Enhances Blood Amino Acid Concentrations Similar to Soy Protein in A Human Trial
Source: Nutrients. 2018 Sep 22;10(10):1357. doi: 10.3390/nu10101357 (PMC6212924; doi:10.3390/nu10101357)
Supplement: Supplementary file 1 [file nutrients-10-01357-s001.pdf]

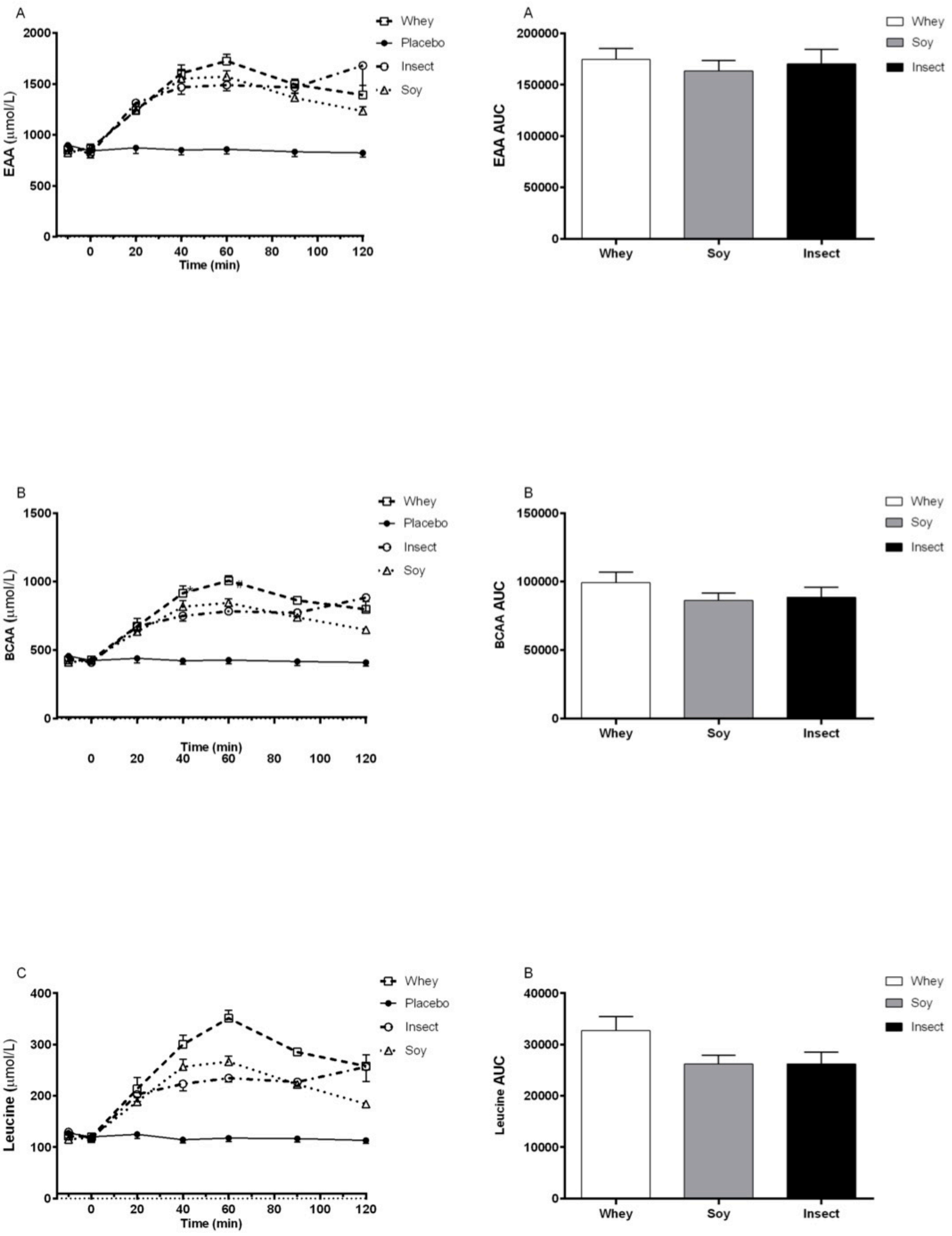

Figure 5. Adjusted blood amino acid concentrations of essential amino acids (EAA), branched-chain amino acids (BCAA) and Leucine after ingestion of whey, soy or insect protein isolate. Data is adjusted as AA concentration per served gram of given protein corresponding to the evaluated total amino acid concentration as shown in Table 2 (Whey: 27.6 g, Soy: 23.2 g, Insect: 21.1 g). All values are mean ± SD; *n* = 6 per group.
